# Immunomodulatory Effects of 17*β*-Estradiol on Epithelial Cells during Bacterial Infections

**DOI:** 10.1155/2018/6098961

**Published:** 2018-08-29

**Authors:** Ivan Medina-Estrada, Nayeli Alva-Murillo, Joel E. López-Meza, Alejandra Ochoa-Zarzosa

**Affiliations:** ^1^Trayectoria en Genómica Alimentaria, Universidad de la Ciénega del Estado de Michoacán de Ocampo, Sahuayo De Morelos, MICH, Mexico; ^2^Departamento de Biología, División de Ciencias Naturales y Exactas, Universidad de Guanajuato, Guanajuato, GTO, Mexico; ^3^Centro Multidisciplinario de Estudios en Biotecnología, Facultad de Medicina Veterinaria y Zootecnia, Universidad Michoacana de San Nicolás de Hidalgo, Morelia, MICH, Mexico

## Abstract

The innate immune system can function under hormonal control. 17*β*-Estradiol (E2) is an important sexual hormone for the reproductive cycle of mammals, and it has immunomodulatory effects on epithelial cells, which are the first line of defense against incoming bacteria. E2 regulates various pathophysiological processes, including the response to infection in epithelial cells, and its effects involve the regulation of innate immune signaling pathways, which are mediated through estrogen receptors (ERs). E2 modulates the expression of inflammatory and antimicrobial elements such as cytokines and antimicrobial peptides. The E2 effects on epithelial cells during bacterial infections are characterized by an increase in the production of antimicrobial peptides and by the diminution of the inflammatory response to abrogate proinflammatory cytokine induction by bacteria. Here, we review several novel molecular mechanisms through which E2 regulates the innate immune response of epithelial cells against bacterial infections.

## 1. Introduction

17*β*-Estradiol, also known as E2 (due to its two hydroxyl groups), is a steroidal hormone derived from cholesterol and is the most predominant and potent sexual hormone during the reproductive stage of females [1]. The principal effects of E2 are associated with reproductive and sexual functions, although it is also involved in the development of different pathologies, such as cancer, autoimmune diseases, and infectious processes, where the innate immune response (IIR) can be altered by this hormone [2].

The main immunomodulatory effects of E2 have been described in the epithelium of the reproductive tract of females, where it modulates the IIR helping to maintain the microbiota in addition to favoring an anti-inflammatory environment [3–5]. E2 can also regulate the differentiation and proliferation of epithelial cells in several tissues (e.g., epithelial mammary cells) [6, 7]. The anti-inflammatory properties of E2 have been demonstrated in different epithelial models, such as the human uterus, rat oviduct, and mouse intestine, and it is well known that E2 can reverse the inflammatory response induced by bacterial components or whole pathogens. In addition, in some models, it has been reported that E2 improves the antimicrobial response against different pathogens inducing the production of antimicrobial peptides. To date, there is little information concerning the immunomodulatory effects of E2 in epithelial cells during infections [3, 8, 9]. Thus, the objective of this review was to provide the current knowledge on the pathways and elements that participate in the immunomodulatory effects of E2 in epithelial cells during bacterial infections.

## 2. General Aspects of E2

**Figure 1:**
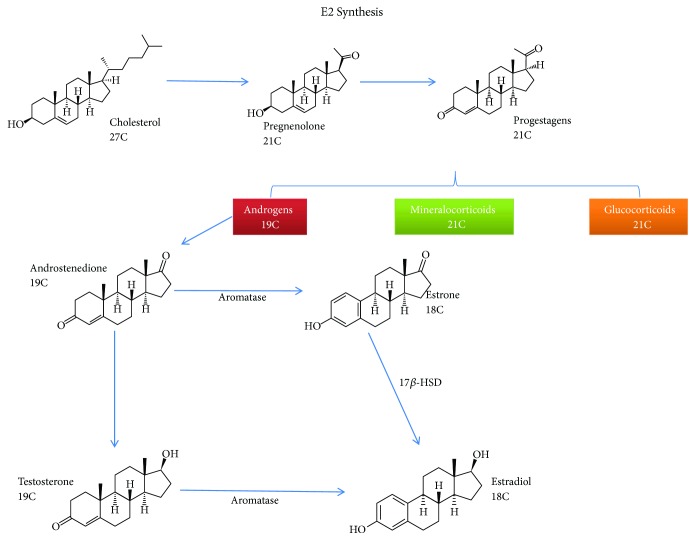
Estradiol synthesis. The production of estradiol can occur in different cell types. Estrogen is a product derived from cholesterol by a series of reactions throughout estrogen biosynthesis. The aromatase enzyme participates in the last step in E2 synthesis. Aromatase is a member of the cytochrome P450 superfamily and is widely expressed in many cell types. As a result of these reactions, an 18-carbon molecule is produced with two hydroxyl groups in its molecular structure. The resulting molecule also contains an aromatic ring with a hydroxyl group on carbon 3 and a *β*-hydroxyl group or ketone at position 17 of ring D. The phenolic ring A is the main structural feature in which selective and high-affinity binding to estrogen receptors occurs [10, 23].

E2 is the most abundant and potent natural estrogen in all vertebrates. E2 is a C18 steroid derived from cholesterol and is the main estrogen present in the serum of mammal females at reproductive age. In humans and other vertebrates, estrogens are made primarily in the female ovaries, but small amounts of this hormone may be produced in the male testes as well as in the adrenal glands, brain, and fat tissue of both sexes. E2 synthesis involves several chemical intermediates, such as progestagens and androgens (e.g., testosterone), which are derived from cholesterol (Figure 1). Testosterone undergoes conversion to estradiol by aromatase (P450aro = CYP19), resulting in the loss of one carbon (C19) and the aromatization of the A-ring, thereby producing E2. An alternative pathway to synthesize E2 occurs through the conversion of androstenedione to estrone (via aromatase), which can be enzymatically converted to E2 [10].

The main targets of E2 are the reproductive female organs (ovary, uterus, and mammary gland). E2 acts in a vast variety of cell populations; however, its main targets are epithelial cells. This hormone regulates epithelial cell differentiation, proliferation, and apoptosis, which are processes intimately linked to the female reproductive cycle such as lactation or ovulation [11–13]. E2 functions are diverse and play important roles in the regulation of metabolism (body fat distribution), hemodynamics (vascular tone), reproduction (ovulation and development of primary and secondary sexual characters, including growth and involution of the mammary gland), and pathological processes, such as cancer and diseases, where the immune response is compromised [11, 14, 15].

### 2.1. Estrogen Receptors

The effects of E2 are mediated by estrogen receptors (ERs), which are classified as ER*α* (565 aa) and ER*β* (530 aa), and these receptors were characterized in 1973 for the first time in a rat uterus [16]. ERs belong to the superfamily of nuclear receptors, functioning as transcriptional factors or associating with other transcriptional factors to regulate the expression of many genes related to the functions described for E2 [17]. In addition to nuclear receptors, there is a third ER, namely GPR30, which belongs to the G protein-coupled receptor family. The GPR30 receptor is located in the endoplasmic reticulum and plasma membrane and is mainly associated with nongenomic responses [16, 18].

The nuclear receptors ER*α* and ER*β* have well defined the functional and structural subunits. Both ERs remain inactive when they are bound to the heat shock protein complexes Hsp70 and Hsp90; however, binding with their ligands (e.g., E2) induces a conformational change, which favors the dissociation of the ER complex and promotes ER activation and translocation to the nucleus. In the nucleus, ER interacts with estrogen response elements (EREs) present in the DNA sequence (the minimum consensus sequence is a palindromic repeat sequence of 5′-GGTCAnnTGACC-3′). EREs are present in many gene promoters such as oxytocin, *c-fos*, *c-myc*, TGF-*α*, prolactin, complement proteins, and lactoferrin [19]. ERs can also be activated independent of estrogen binding (nonclassical signaling). This kind of ER activation is the consequence of several posttranslational modifications (phosphorylation, acetylation, and methylation) induced by different enzymes [20].

E2 levels vary depending on the species and its reproductive stage. The range in mammal females oscillates between 50 pg/ml (standard for basal condition) and 1500 pg/ml (standard for the preovulatory phase), which is associated with the effects that E2 have on reproduction and is the reason why sexual hormone levels are varied in mammals [21, 22]. ERs as well as E2 have been associated with some pathologies, such as cancer, being breast cancer the principal disease in which ERs are involved [23, 24]. Nevertheless, ERs and E2 also play an important role in the IIR to different stimuli. For example, females are more susceptible to develop inflammatory lung conditions and sex hormones have been implicated in this process [25]. However, the immune mechanisms responsible for sex-based disparity are unknown. The data suggest that specific sex hormone influences the IIR [1]. Accordingly, ERs can modulate the activation of transcriptional factors associated with the IIR, such as NF-*κ*B, SP1, AP-1, and C/EBP*β*. As a consequence, ERs regulate the transcription of elements of the inflammatory response, such as proinflammatory cytokines, anti-inflammatory cytokines, antimicrobial peptides, and chemokines. In addition, in response to their ligands, ERs regulate different signaling pathways, which can also be associated with the IIR [26].

GPR30 is associated with cellular estrogen functions, including canonical genomic signaling and novel nongenomic responses. The nongenomic events include production of second messengers (Ca^2+^ and cAMP), activation of tyrosine kinase receptors (such as EGFR and IGF-IR), and activation of different protein/lipid kinase pathways (PI3K, Art, MAPK, Scr, and PKA/PKC) [16]. Thus, GPR30 signaling pathways are also related to the regulation of the IIR. However, the information concerning these functions is scarce.

## 3. Hormonal Regulation of the Innate Immune Response

Diverse hormones have been reported to participate in the modulation of the IIR, which is activated by different stimuli such as those occurring in infectious diseases or stress conditions, which can modulate the inflammatory response. This response during infections is mainly characterized by the production of proinflammatory cytokines, namely tumor necrosis factor alpha (TNF-*α*), interleukin-1 beta (IL-1*β*), interleukin-2 (IL-2), and interleukin-6 (IL-6), as well as chemokines. These molecules are secreted by the injured tissue as well as by innate cells such as macrophages, dendritic cells, and mast cells in order to recruit neutrophils and monocytes to the affected tissue. Through the release of antimicrobial molecules and active phagocytosis, innate immune cells act to eliminate the foreign agent and recover tissue homeostasis [27]. Sexual hormones play an important role during the IIR to infection in particular estradiol, progesterone, and androgens [28].

On the other hand, the stress response stimulates the hypothalamic-pituitary-adrenal (HPA) axis, inducing the release of the corticotrophin-releasing hormone (CRH) from the hypothalamus to the pituitary portal system. Corticotrophin is then released from the pituitary gland to the systemic circulation, stimulating the suprarenal cortex to reliberate glucocorticoids [29]. At the same time, stress induces the activation of the sympathetic nervous system, which leads to catecholamine release from the autonomic nervous terminals and the adrenal medulla [30]. Glucocorticoids and catecholamines have well-known immunoregulatory functions, namely, reducing the inflammatory response activated by the infection, which leads to the production of anti-inflammatory cytokines [29].

In addition to the HPA axis, other hormones have been implicated in the regulation of the IIR. Indeed, receptors for many hormones are expressed in cells from the innate and adaptive immune systems, which is the case for prolactin, growth hormone, thyroid hormone, or estrogen. The immunomodulatory effects of the hormones depend on the type of tissue, the hormone concentration, the physiological context, or the activated signaling pathway [31].

With regard to estradiol, its immunomodulatory properties comprise both the development as well as the function of innate immune cells. In this sense, ERs are expressed in immune cells triggering different signaling pathways. Estradiol exhibits both proinflammatory and anti-inflammatory responses related to hormone concentrations [26].

The transition to menopause, characterized by declining estradiol levels, has an impact on immunosenescence increasing the susceptibility to infection diseases and decreasing the efficacy of vaccination. These effects in postmenopausal women are characterized by an upregulation in the production of the cytokines TNF-*α*, IL-1*β*, IL-10, and IL-6, the stimulation of the cytotoxic activity of NK cells, and a diminished phagocytic capacity of dendritic cells (DCs), which leads to impaired antigen presentation and activation of the adaptive immune system. Upon hormone replacement therapy, these age-related changes can be partially reverted [32]. In addition, women are susceptible to develop postmenopausal pathologies, such as diabetes, atherosclerosis, and cardiovascular diseases, all of them characterized by inflammatory hallmarks. Epidemiological studies worldwide suggest that hormonal therapy, which mainly consists of E2 administration (through different routes and hormonal combinations), can revert or ameliorate these inflammatory conditions. However, there are contradictory results and further research is necessary in order to clarify the effects of hormone replacement therapy in the development of inflammatory diseases [33].

### 3.1. Roles of Epithelial Cells during the Innate Immune Response

Mucosal epithelial cells display an autonomous IIR against invading bacteria and are able to discriminate between pathogen and nonpathogen microorganisms, thereby generating an inflammatory response against pathogens. This discrimination can be achieved through different mechanisms. For example, mucosal epithelial cells are covered by a film of thick mucus layer that protects epithelium from unwanted microorganisms. The mucus layer serves as matrix in which antimicrobial peptides produced by the epithelial lining are embedded [34]. However, many bacterial species invade epithelial cells to escape from antimicrobial substances or the immune system and they are able to adhere and invade epithelia [35].

Bacteria express molecules that can be recognized by the innate immune system, and these elements are known as pathogen-associated molecular patterns (PAMPs) [36]. PAMPs are recognized by transmembrane and cytoplasmic receptors that are expressed by a broad range of cell types, including epithelial cells. These receptors are known as pathogen-recognizing receptors (PRRs), such as Toll-like receptors (TLRs) and Nod-like receptors (NLRs), or coreceptors, such as CD14 and CD16 [37]. Even though being a “classic” defense, cells such as macrophages, neutrophils, and dendritic cells have a great quantity and different types of these receptors. Epithelial cells also express these receptors and are effective against infections. The ligands for these types of receptors are varied and include fungal or bacterial membrane constituent molecules or viral or bacterial genetic material [35]. There is evidence supporting that PAMPs from symbiotic microorganisms are less agonistic to PRRs than those of pathogenic microorganisms inducing immune tolerance or physiological inflammation, which maintains a significant proportion of resident macrophages and dendritic cells in a situation of immaturity, and of a proper balance between regulatory T cell (Treg) lymphocytes and inflammatory lymphocytes (Th1 and Th17 T) [34].

**Figure 2:**
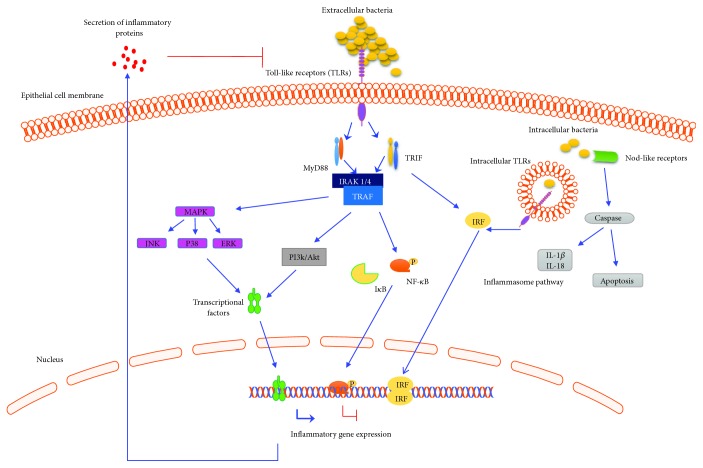
Signal transduction pathways of epithelial cells involved in the recognition of pathogenic bacteria. Epithelial cells are part of the innate immune system, which senses the presence of microbial pathogen-associated molecular patterns (PAMPs) present in bacteria. These elements activate inflammatory pathways mainly through the activation of Toll-like receptors (TLRs). TLR proteins are coupled to the scaffold proteins, myeloid differentiation primary response 88 (MyD88), or TIR domain-containing adapter-inducing interferon-*β* (TRIF), which are associated with interleukin-1 receptor-associated kinase 1/4 (IRAK1/4). This kinase is coupled to TNF receptor-associated factor (TRAF) [38]. TLR signaling results in the downstream activation of the following three major families of proteins important in activating inflammatory gene expression: interferon regulatory factors (IRFs); mitogen-activated protein kinase (MAPK) pathway, such as c-Jun N-terminal kinase (JNK), protein 38 (P38), and extracellular signal-regulated kinases (ERKs); and the canonical inflammatory pathway, namely, nuclear factor kappa-light-chain-enhancer of activated B cells (NF-*κ*B). However, other transcriptional factors, such as activator protein 1 (AP-1), can also be activated. Bacteria induces the expression of many inflammatory genes and cytokine secretion, such as interleukin 1-beta (IL-1*β*), interleukin-6 (IL-6), and tumor necrosis factor-alpha (TNF-*α*) [36]. Intracellular bacteria can be recognized in epithelial cells by TLRs (endosome) or Nod-like receptors (NODs), which can activate the inflammasome. This is a multiprotein complex activated by caspases, and its activation and composition depend on the activator, which initiates inflammasome assembly, resulting in the processing of inflammatory cytokines such as IL-1*β* and interleukin 18 (IL-18) [63].

The recognition of PAMPs from pathogens triggers signaling pathways and, as a consequence, activates different transcriptional factors, which regulate cytokine, chemokine, or antimicrobial peptide gene expression and protein secretion; all of these elements are associated with the IIR. The objective of this response is to eliminate the pathogen and repair the damage that it caused (Figure 2). The most commonly activated elements after the recognition of pathogens are as follows: members of MAPKs, transcriptional factors, including NF-*κ*B, AP-1, and SP1; proinflammatory cytokines, such as TNF-*α*, IL-1*β*, and IL-6; anti-inflammatory cytokines, including IL-10; antimicrobial peptides; and oxygen- and nitrogen-reactive species [38]. In Figure 2, a general overview of the signaling inflammatory events of epithelial cells during bacterial infections is shown.

## 4. Functions of E2 in Epithelial Cells

In general, the effects of E2 on epithelial cells activate the classic genomic pathway, which occurs over the course of hours. E2 binding to ER induces some conformational changes allowing ER to dissociate from chaperone heat-shock proteins and dimerize with other receptors (ERs). This complex binds directly either to an ERE in target gene promoters or to transcriptional factors via protein tethered to DNA [11]. In contrast, nongenomic signaling via E2-ERs occurs quickly (minutes or seconds). The ligand-receptor complex can also interact with G proteins, growth factor receptors, or tyrosine kinases, thus facilitating the interaction and rapid intracellular signaling [16].

Both classic and nongenomic E2-ER signaling pathways lead to a wide variety of biological cell functions in different epithelia. The classic effects of E2 on epithelial cells are associated with proliferation, differentiation, and cellular apoptosis. For example, the epithelial cells of mammary glands—one of the E2 target tissues—are exposed to major morphological and biochemical changes during the lactation cycle [12]. Additionally, steroid hormones of the ovary and placenta have been implicated as stimulators of mammary gland development, involving complex interactions between E2 and epithelial mammary cells, resulting in mammogenesis, lactogenesis, galactopoiesis, and involution [39]. The genomic biological responses of E2 in mammary glands are predominantly mediated by ER*α*, which is localized in epithelial compartments. Mammary proliferation depends on the nature of the reproduction cycle. In many species, duct extension and branching are followed by growth of alveoli lobules. During milk production, basal levels of E2 are necessary to maintain metabolism and several specific hormone actions and they are also expected to play a permissive role during lactation. After lactation, the mammary gland has a gradual involution. The induction of apoptosis mediated by E2 (1 × 10^−7^ mol/ml) is necessary for this process, which requires ER*α* at 2–4 weeks of involution and ER*β* at 2–4 weeks after this event [13]. For all of the E2 effects described, different factors associated with E2-ER signaling pathways are involved, such as epidermal growth factor (EGF), TGF-*α*, insulin-like growth factor (ILG-F), and TNF-*α*. These elements modulate the survival and apoptosis of mammary epithelial cells at 10^−10^ mol/ml E2 [40].

The female reproductive tract (epithelial cell specificity) is exposed to E2 effects, which influence processes associated with reproduction and immunity [4]. The vagina, cervix, uterus, oviduct, and ovaries are strongly influenced by E2. This hormone increases the proliferation of epithelial cells in both the uterus and the vagina. For example, it has been observed in mouse uterus that after the preovulatory E2 surge, early (within 6 h) morphological and biochemical changes occur including vascular permeability, hyperemia, prostaglandin release, glucose metabolism, eosinophil infiltration, and lipid and protein synthesis [41]. In addition, E2 induces ER*α* recruitment to ERE sites in target genes of mouse uterus, which leads to RNA and DNA syntheses, epithelial cell proliferation, and their differentiation toward a columnar secretory epithelium [42]. These effects are achieved at long time periods (after 24–72 h).

Otherwise, vaginal epithelial cells respond to E2 by undergoing cornification (production of keratins and involucrin), a process that involves both proliferation and differentiation. These effects are mediated by ER*α* in a direct way as well as through a paracrine route (involving stroma cells) [43].

E2 also modulates the permeability of the lower female reproductive tract (vagina and ectocervix). Epithelial cells are linked by tight junction proteins, regulating the traffic of molecules across the epithelium. In the lower female reproductive tract, the stratified squamous epithelium shows tight junctions between basal epithelial cells. E2 increases the relaxation of epithelial tight junctions, which induces the flux across the epithelium. These effects are mediated by the expression of claudin and occludin [9, 44].

E2 also promotes lactobacillus growth in vaginal epithelial cells by increasing the storage of glycogen in the suprabasal and apical layers [45]. Glycogen is a substrate for acid production by these bacteria maintaining a low-pH environment [7]. Epithelial cells from the urinary tract (bladder epithelium) are also influenced by E2, and these cells play an important role of protection from infectious diseases where E2 has a relevant function, increasing the production of antimicrobial peptides and tightening intercellular connection, thereby preventing bacteria to reach the cells where they can hide and cause infection [7]. *In vitro* and *in vivo* studies indicate that the production of different antimicrobial peptides increases in bladder epithelial cells after E2 treatment (see below).

In addition, it has been shown in rabbit bladders that E2 increases the volume of smooth muscle cells as well as vascularization, promoting bladder contraction [46]. In the absence of E2, the residual urine increases, whereas the urine flow is reduced, impairing the mechanical clearance of bacteria [47].

### 4.1. Immunomodulatory Effects of E2 in Epithelial Cells

The epithelial IIR is fast and acts directly upon the pathogen attack or tissue damage [48]. Several studies have demonstrated the regulation of the IIR of epithelia by E2. In all of the epithelial models where E2 plays an immunomodulatory role, E2 has also been demonstrated to have anti-inflammatory effects. In human epithelial cells from the endometrium, E2 (10^−7^ M) shows an anti-inflammatory action reversing the effects caused by PAMPs, such as Poly I:C [49]. In addition, E2 can block the inflammatory effects caused by IL-1*β* and lipopolysaccharide (LPS) from *Escherichia coli* in human and rat uterus [6, 49]. In bovine oviduct, LPS stimulation increases the secretion of IL-1*β* and TNF-*α* and it induces the expression of TLR2 and TLR4. However, E2 (1 ng/ml) reverses these inflammatory effects [44]. In the same way, the production of nitric oxide induced via mechanical damage or LPS stimulation in rat intestinal epithelium is diminished by E2 [3, 50].

Despite the aforementioned evidence, the relation of E2 with the IIR during infectious processes has not been properly analyzed because epithelial cells are challenged with PAMPs or proinflammatory cytokines instead of whole bacteria, in most of the models.

Experimental evidence suggests that E2 performs its immunomodulatory effects through the regulation of TLRs because they are the main PRRs that trigger the IIR signaling pathways. E2 (10^−8^ M) through ER*α* modulates the signaling of TLR3 (which recognizes double-stranded RNA) in the human reproductive tract during the beginning of the gestational cycle. Interestingly, the increase of E2 reduces the abundance of TLR3 favoring an anti-inflammatory environment [51].

**Figure 3:**
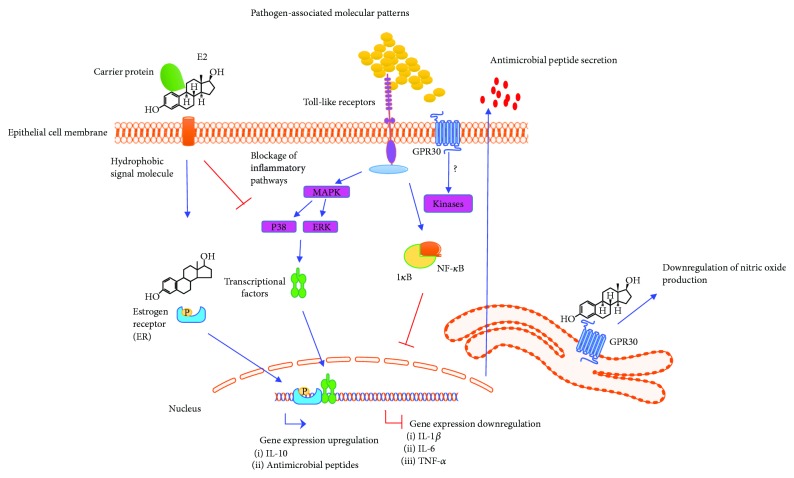
Anti-inflammatory and antibacterial effects of E2 in epithelial cells. E2 mediates its effects in epithelium by binding to intracellular receptors (ERs), leading to the translocation of hormone-ER (ER phosphorylate) complexes into the nucleus where they modulate gene transcription through binding to estrogen-responsive elements (EREs) on DNA (5′GGTCAnnnTGACC-3′ consensus sequence). There is another type of ER, namely, G protein-coupled receptor 30 (GPR30), which binds E2, tamoxifen, and ICI 182780 (Fulvestrant). This receptor promotes the activation of epidermal growth factor receptor (EGFR) and mediates signaling in breast, endometrial, and thyroid cancer cells. This receptor activates signaling pathways in the cytosol and at the plasma membrane [18]. However, most of the molecular targets activated by GPR30 located at the plasma membrane during infections are unknown. GPR30 also has effects on the IIR associated with nongenomic responses (e.g., nitric oxide production). When cells are infected by bacteria, they trigger the activation of proinflammatory signaling pathways, mainly depending on TLRs. These pathways (described above) promote the production of proinflammatory elements [16]. However, E2 can modulate these pathways, influencing the IIR and decreasing the proinflammatory elements by blocking the activation of transcriptional factors, such NF-*κ*B (canonical inflammatory pathway), ultimately resulting in the downregulation of IL-1*β*, IL-6, and TNF-*α* gene expression. In contrast, the E2-ER complex mainly induces the gene expression of AMPs, such as defensins, and promotes an anti-inflammatory environment by inducing interleukin-10 (IL-10) gene expression. Gene expression regulated by E2 depends on the concentrations of the hormone and the interaction with pathogenic bacteria. It is well known that E2 reverses the inflammatory effects (association with bacteria and proinflammatory cytokines) and promotes an anti-inflammatory environment [8, 60, 64, 65].

E2 also regulates different inflammatory transcriptional factors. For example, in rat aortic smooth muscle cells, E2 inhibits the activation of NF-*κ*B, thus avoiding its dissociation from the I*κ*B inhibitor [52]. In addition, it is known that in both human uterine and urinary epithelial cells, E2 can modulate the activation of other transcriptional factors such as AP-1, SP1, STAT, and vitamin D receptor (VDR), which also participate in the regulation of the IIR. In this sense, E2 inhibits STAT phosphorylation and promotes the activation of VDR, modulating the MAPK signaling pathway (Figure 3) [6, 7, 53]. Finally, in human endometrium, E2 (10^−8^ M or 10^−9^ M) inhibits the protein levels of the IL-1*β* receptor, thus reversing the stimulatory effects of IL-1*β* on mRNA expression of TNF-*α*, IL-8, and NF-*κ*B [6]. A summary of the effects of E2 on the IIR of epithelial cells is shown in Figure 3.

### 4.2. Antimicrobial Activity of E2 against Bacterial Infections in Epithelial Cells

Antimicrobial peptides (AMPs) are a diverse group of molecules with a broad range of activities, including cytotoxic, antifungal, antibacterial, or immunomodulatory effects. In mammals, these molecules are produced by different types of cells, highlighting those that are in intimate contact with pathogens such as epithelial cells [54]. In different models of infection, E2 exerts antibacterial activity against many pathogens because it favors the expression and secretion of AMPs. Human beta defensin-2 (HBD-2) is one of the most relevant AMPs in the human reproductive tract, and its secretion is regulated by E2. For example, human uterine epithelium exposed to LPS in the presence of E2 (10^−3^ M) shows increased secretion of the AMPs, calcineurin-like metallophosphoesterase superfamily protein (SLP1), and HBD-2, as well as decreased production of proinflammatory cytokines via NF-*κ*B inhibition [3]. Moreover, in vaginal epithelium, E2 (2 nM) induces HBD-2 secretion in response to LPS [55]. In addition, growth inhibition of *S. aureus* in the presence of E2 has been reported in a rat uterus and this inhibition is attributed to AMP secretion in the uterine lumen [8]. In the urinary tracts of women treated with E2 (10^−3^ M), a lesser recurrence of *Escherichia coli* infections has been observed, which is associated with the production of AMPs, such as defensins (HBD-1 and HBD-2), and the homeostasis of the common microbial flora [3]. In similar models (postmenopausal women treated with estrogens), a diminished risk of bacterial infections has been observed due to the increase of mRNA coding for the AMPs HBD-1 and HBD-3, as well as psoriasin and cAMP [3, 6, 8].

During the menstrual cycle, the fluctuation of hormonal concentrations leads to immune changes in the female reproductive tract (FRT). These changes vary depending on the location (upper or lower FRT) and the hormonal concentrations, which fluctuate differentially across the tissues in relation to their levels in circulation. At mid-cycle when E2 levels are high, there is a window of vulnerability for the establishment of infections in the lower FRT (viral, bacterial, or fungal) as a result of the inhibition in the production of AMPs (such as HBD-2). By contrast, in the upper FRT (uterus) persists a reduced activity of cytotoxic cell at a time when the production of AMPs is enhanced optimizing the conditions for a successful implantation [9]. In addition, during the proliferative stage of the menstrual cycle, cervical/vaginal secretion contains higher levels of other AMPs in relation with other stages of the cycle (medium and secretory phases), for example, the secretory leukocyte protease inhibitor-1 (SLP1), HBD-1, human neutrophil proteins 1–3 (HNP1–3), or lactoferrin [5, 6, 56, 57].

In animal models, immunomodulatory effects of E2 also comprise the production of AMPs. For example, SBD-1 (sheep beta-defensin-1) defensin is induced in ovine oviduct epithelial cells by E2 (10^−8^ M) [5]. In male rabbits exposed to *S. aureus* (a strain causing toxic shock), E2 treatment protects against the infection [58]. Similarly, in rat uterus using *in vivo* and *in vitro* experiments, higher levels of E2 induce resistance against *E. coli* infection and this effect is attributed to the secretion of antimicrobial molecules [8]. In human bladder epithelial cells, E2 induces the production of defensins HBD1-3, ribonuclease (RNase) 7, psoriasin, and cathelicidin; these effects can be associated with a reduced *E. coli* infection [59]. These findings indicate that AMP production constitutes a relevant defense mechanism induced by E2 in epithelia.

**Figure 4:**
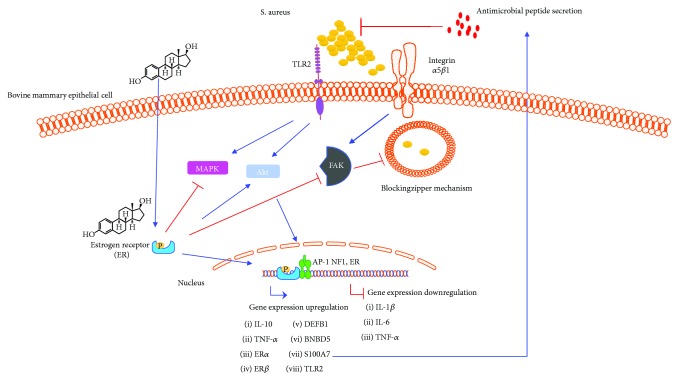
Effects of E2 on bovine mammary epithelial cells during *Staphylococcus aureus* infection. During *S. aureus* infection in bovine mammary epithelial cells (bMECs), E2 inhibits the mitogen-activated protein kinases (MAPKs), resulting in anti-inflammatory effects (decreasing gene expression of IL-1*β* and IL-6 as well as increasing gene expression of IL-10). E2 also actives protein kinase B (Akt), which in turn activates transcriptional factors, such as activator protein 1 (AP-1), NF1, or ER, that are related to IIR gene expression, such as AMPs, IL-10, or TNF-*α*. Through the zipper mechanism, *S. aureus* is internalized into epithelial cells, preventing it from being detected by the defense system of the host cell. However, E2 can block the signaling pathway of the zipper mechanism through the inhibition of focal adhesion kinase (FAK), resulting in the reduction of *S. aureus* internalization. The production of antimicrobial molecules (e.g., antimicrobial peptides) by bMECs treated with E2 also reduces *S. aureus* viability. However, the identity of the AMPs secreted by bMECs remains unknown. By RT-qPCR, the upregulation in the gene expression of DEFB1 (defensin beta 1), BNBD5 (bovine neutrophil beta-defensin 5), and S100A7 (psoriasin) has been shown. E2 induces the expression of TLR2, a key receptor in the recognition of *S. aureus*, as well as upregulates the expression of its receptors (ER*α*/*β*) [54, 58, 60].

Evidence from our group indicates that bovine mammary epithelial cells (bMECs) treated with E2 produce antimicrobial molecules (probably AMPs) as the conditioned medium of bMECs treated with E2 inhibits the viability and growth of *S. aureus*. In addition, bMECs treated with E2 show upregulated gene expression of antimicrobial molecules, such as DEFB1 (defensin beta 1), BNBD5 (bovine neutrophil beta-defensin 5), and S100A7 (psoriasin) [60]. These effects are achieved through the activation of transcriptional factors, such as AP-1, NF1, or ER (data unpublished) (Figure 4). These events lead to the inhibition of *S. aureus* internalization, presumably through the inhibition of focal adhesion kinase (FAK) (Figure 4) [60].

Considering the roles of epithelial cells during the IIR (Section 3.1), the combination of an anti-inflammatory environment and the antimicrobial response is essential for the maintenance of homeostatic epithelial flora. During infections, the host initiates an inflammatory response, sometimes aggravated, which results in damage to the tissue. Nevertheless, E2 in epithelial cells from all of the models described (using PAMPs or whole pathogens) inactivates inflammatory elements but activates the production of antimicrobial molecules, presumably induced via AP-1 or SP-1, thereby favoring elimination of the pathogen. In addition, the production of AMPs by the host can enhance the epithelial inflammatory response accordingly to reports that show the immunomodulatory properties of these molecules through the induction of chemotaxis, enhancing the phagocytic activity of macrophages and dendritic cells, upregulating the production of inflammatory cytokines, chemokines, and so on [54, 61]. Furthermore, it is important to highlight that the production of AMPs is not enough to counteract an infection, in particular infections caused by intracellular pathogens, which require of a correct inflammatory response [62].

## 5. Concluding Remarks and Future Directions

Epithelial cells constitute a fundamental part of the local inflammatory response and host defense against pathogens. Epithelium is a target of E2 effects where the hormone induces the production of antimicrobial molecules, thereby reinforcing its role as a defense barrier against microorganisms besides its immunomodulatory properties. In addition, AMPs can also enhance the IIR through the regulation of other immunomodulatory effects.

Antimicrobial and anti-inflammatory properties of E2 are highly relevant along the reproductive life of women. For example, in the middle of the menstrual cycle when E2 levels are high, women are more likely to experience infections in the lower FRT (viral, bacterial, or fungal) as consequence of reducing the production of AMPs by epithelial cells. These effects of E2 are selective in ways that vary according to its concentrations and the FRT site. Based on epidemiological data, women appear to lose their immunological advantage after menopause, since in aging, they are more susceptible to infections and to develop inflammatory diseases. However, the participation at these stages of the E2-induced antimicrobial response requires further research.

Results discussed in this review may lead to the development of new hormonal strategies that would improve and enhance the IIR of epithelial cells against infections at the different reproductive stages in women.
